# The use of personalized biomarkers and liquid biopsies to monitor treatment response and disease recurrence in locally advanced rectal cancer after neoadjuvant chemoradiation

**DOI:** 10.18632/oncotarget.5256

**Published:** 2015-10-06

**Authors:** Paola Carpinetti, Elisa Donnard, Fabiana Bettoni, Paula Asprino, Fernanda Koyama, Andrei Rozanski, Jorge Sabbaga, Angelita Habr-Gama, Raphael B. Parmigiani, Pedro A.F. Galante, Rodrigo O. Perez, Anamaria A. Camargo

**Affiliations:** ^1^ Ludwig Institute for Cancer Research, São Paulo, SP, Brazil; ^2^ Centro de Oncologia Molecular Hospital Sírio Libanês, São Paulo, SP, Brazil; ^3^ Programa de Pós Graduação em Bioquímica, Instituto de Química, Universidade de São Paulo, SP, Brazil; ^4^ Centro de Oncologia Clínica, Hospital Sírio Libanês, São Paulo, SP, Brazil; ^5^ Angelita & Joaquim Gama Institute, São Paulo, SP, Brazil; ^6^ University of São Paulo, School of Medicine, São Paulo, SP, Brazil

**Keywords:** rectal cancer, liquid biopsies, ctDNA, personalized biomarkers, neoadjuvant therapy

## Abstract

Neoadjuvant chemoradiotherapy (nCRT) followed by surgery is the mainstay treatment for locally advanced rectal cancer. Variable degrees of tumor regression are observed after nCRT and alternative treatment strategies, including close surveillance without immediate surgery, have been investigated to spare patients with complete tumor regression from potentially adverse outcomes of radical surgery. However, clinical and radiological assessment of response does not allow accurate identification of patients with complete response. In addition, surveillance for recurrence is similarly important for these patients, as early detection of recurrence allows salvage resections and adjuvant interventions. We report the use of liquid biopsies and personalized biomarkers for monitoring treatment response to nCRT and detecting residual disease and recurrence in patients with rectal cancer. We sequenced the whole-genome of four rectal tumors to identify patient-specific chromosomal rearrangements that were used to monitor circulating tumor DNA (ctDNA) in liquid biopsies collected at diagnosis and during nCRT and follow-up. We compared ctDNA levels to clinical, radiological and pathological response to nCRT. Our results indicate that personalized biomarkers and liquid biopsies may not be sensitive for the detection of microscopic residual disease. However, it can be efficiently used to monitor treatment response to nCRT and detect disease recurrence, preceding increases in CEA levels and radiological diagnosis. Similar good results were observed when assessing tumor response to systemic therapy and disease progression. Our study supports the use of personalized biomarkers and liquid biopsies to tailor the management of rectal cancer patients, however, replication in a larger cohort is necessary to introduce this strategy into clinical practice.

## INTRODUCTION

Neoadjuvant chemoradiotherapy (nCRT) has become the preferred treatment for patients with locally advanced rectal cancer (cT3–4 or cN+), leading to significant decrease in tumor size (downsizing) and a shift towards earlier disease stage in the primary tumor and lymph nodes (downstaging) [1]. However, the response of individual tumors to nCRT is not uniform; some patients have complete eradication of the tumor, while others present variable degrees of tumor regression. Complete pathologic response (pCR) to nCRT can be observed in up to 42% of the patients and has been associated with improved local disease control and overall survival [2].

The observation of complete pathologic response in significant proportion of these patients has led colorectal surgeons to consider alternative treatment strategies to radical surgery based on tumor response to nCRT [3]. Patients with no clinical or radiological evidence of residual disease (complete clinical response) have been offered less aggressive treatment strategies, including close surveillance without any immediate surgery (Watch and Wait Strategy) [4]. This conservative strategy has the advantages of an organ-sparing approach by avoiding significant postoperative morbidity, functional disorders associated with surgery (fecal incontinence, sexual, and urinary dysfunctions) and the need for intestinal stomas [5]. On the other hand, it requires the precise identification of patients with pCR after nCRT and strict follow-up for early detection of local and systemic recurrences, allowing for salvage resections with no oncologic compromise [6].

Unfortunately, clinical and radiological assessment of tumor response to nCRT is still based on imprecise and subjective findings and do not allow the accurate identification of patients with pCR [7]. Indeed, the risk of local recurrence in patients with complete clinical response with no immediate surgery may be significant [6]. For these reasons, surgical resection after nCRT is still regarded as the cornerstone of curative therapy [8]. In this context, a more precise assessment of tumor response after nCRT would allow the selection of patients with pCR that could be spared from potentially unnecessary radical proctectomy and managed with the Watch & Wait Strategy. Similarly, early detection of local and systemic recurrences would allow salvage resection and adjuvant interventions, significantly affecting oncological outcome [6, 9].

Circulating DNA fragments, carrying tumor-specific genetic alterations (circulating tumor DNA–ctDNA), are shed into the bloodstream by tumor cells undergoing apoptosis or necrosis [10, 11] and the load of ctDNA correlates with tumor staging and prognosis [12]. The detection of ctDNA in the plasma of cancer patients (known as liquid biopsies) has been successfully used to monitor tumor burden and therapy resistance, to evaluate the presence of residual disease after potentially curative treatment and to monitor disease recurrence with high sensitivity and specificity [reviewed in 13, 14]. A challenge for ctDNA analysis is the identification of the tumor-specific mutations to be used as markers. Chromosomal rearrangements - including translocations, insertions, deletions and inversions - are a key feature of tumor genomes, occurring at the earliest stages of tumorigenesis and persisting throughout tumor development [15]. These rearrangements, representing substantial changes of the tumor genome, are not present in the normal cells from cancer patients and have been successfully used to indirectly detect tumor cells. Moreover, since assays developed for the detection of chromosomal rearrangements are more specific and sensitive than those used to detect point mutations, tumor-specific chromosomal rearrangements represent ideal biomarkers for the detection of ctDNA [16].

Indeed, highly sensitive and specific assays developed to detect recurrent chromosomal translocations in hematological tumors have become standard practice to monitor minimal residual disease and predict relapse to targeted therapy, allowing earlier therapeutic managements [17]. Unfortunately, a similar use of chromosomal rearrangements in solid tumors has been hampered until recently by the absence of recurrent rearrangements in these tumors. However, recent advances in sequencing technologies and bioinformatics have enabled the genome-wide identification of patient-specific somatic chromosomal rearrangements in a cost-effective and clinically-relevant timeframe, which can be used as personalized biomarkers for the monitoring of ctDNA [16, 18–20]. In the present study, we report an initial assessment of the use of personalized biomarkers and liquid biopsies for monitoring treatment response to nCRT and detecting residual disease and early recurrence in locally advanced rectal cancer.

## RESULTS

### Characterization of patient-specific chromosomal rearrangements

Four patients were included in this study and pre-treatment characteristics, response to nCRT and follow-up information are presented in Table 1. Mate-pair libraries with insert size ∼ 600pb were generated from tumor genomic DNA and were sequenced using the SOLiD 4.0 sequencing platform. An average of 560 million 50–75bp reads were generated for each of the four tumor samples and were mapped against the human genome reference sequence, resulting in an average of 17Gb of mapped sequences per sample. Sequence and physical coverage varied from 4 to 8.7x and from 13 to 60x, respectively (Supplementary Material Table S3).

**Table 1:** Clinical and pathological information of rectal cancer patients

Patient	Gender	TNM Staging^a^		Local	Response	TRG^b^	Follow-up
		cT	cN				
1	M	3	0	distal	Incomplete	3	Liver metastasis (week 36)^c^
2	F	3	1	distal	Incomplete	2	NED
3	F	3	1	distal	Pathologic complete	4	NED
4	M	3	0	distal	Clinical Complete	-	Liver metastasis (week 62)^c^

Abbreviation: (NED) no evidence of disease

a Tumor staging based on the TNM Classification System

b Tumor regression grade, obtained from the histopathological analysis of the resected tumor, as described in Dworak et. al, 1997.

c Number of weeks after the end of nCRT in which the disease recurrence was detected by clinical and imaging exams.

**Figure 1:**
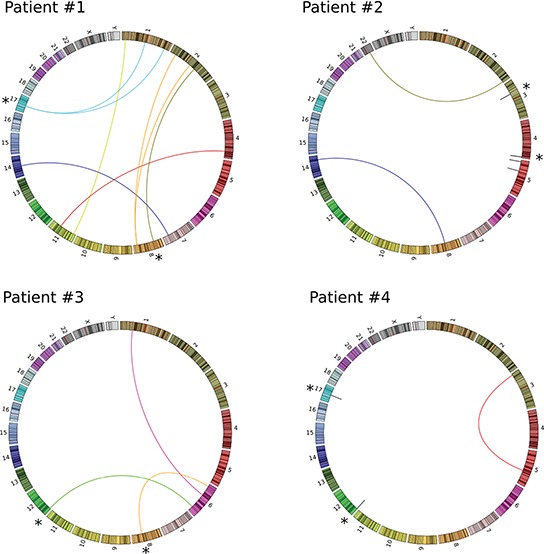
Patient-specific chromosomal rearrangements represented as Circos-plots Chromosome representations are show around the outer cycle and are in a clockwise orientation starting from chromosome 1. Interchromosomal translocations are represented by colored lines linking two chromosomes. Intrachromosomal deletions and inversions are represented by gray lines. * Indicates patient-specific chromosomal rearrangements selected for ctDNA monitoring.

We identified a total of 54 genomic regions containing putative somatic rearrangements in all 4 tumors, with an average of 14 rearrangements per tumor (Supplementary Material Table S4). PCR primers spanning the putative breakpoints were designed to validate the existence and the somatic nature of 29 patient-specific chromosomal rearrangements. Twenty-two of these assays yielded PCR fragments of the expected size when tumor genomic DNA, but not normal DNA, was used as template in the amplification reaction (Supplementary Material Table S5). Sanger sequencing of PCR fragments confirmed the existence of the rearrangement and allowed us to map the breakpoint region at a base pair resolution. All 4 tumor samples had at least 2 bona fide somatic rearrangements that were used as personalized biomarkers for monitoring ctDNA in plasma samples (Figure 1 and Table 2)

**Table 2:** Patient-specific chromosomal rearrangements used for ctDNA monitoring

Patient	SV	Chromosomal Positions^a^				GenomicRegion^b^		Genes^c^
		ChrA	Position	ChrB	Position			
**1**	T01	17	35126102	1	223988874	-	intragenic	*TP53BP2*
**1**	T02	8	40477264	2	152374971	-	intragenic	*ZMAT4, NEB*
**2**	D01	3	60342822	3	60388671	∼45 Kb	intragenic	*FHIT*
**2**	D02	4	185436839	4	185913887	∼500 Kb	intergenic	**−**
**3**	T01	8	93421404	6	13191227	-	intragenic	*PHACTR1*
**3**	T02	12	3607669	6	145470434	-	intragenic	*PRMT8*
**4**	I01	12	1819278	12	−1870423	∼50 Kb	intragenic	*ADIPOR2*
**4**	D02	17	39823076	17	39837384	∼14 Kb	intragenic	*JUP*

Abbreviations: (**SV**) Structural Variation; (**D**) Deletion; (**I**) Inversion; (**T**) Translocation; (**ChrA**) Chromosome A; (**ChrB**) Chromosome B.

a Chromosomal positions were based on human genome reference sequence (hg19), negative signal indicates sequences aligning to the minus strand.

b Genomic Region: approximate size (Kb) of genomic regions involved in deletions and inversions; genomic context base on gene annotation.

c Genes: genes involved in patient-specific chromosomal rearrangements.

### Quantification of ctDNA in the plasma using personalized biomarkers

Serial blood samples were collected prospectively: i) at diagnosis, prior to nCRT initiation, ii) during the resting interval, iii) at the time of clinical evaluation of response and iv) during follow-up for all patients. Using control assays designed to amplify single-copy non-rearranged genomic regions, we confirmed that amplifiable DNA was present in all plasma samples. The amount of total DNA present in the plasma samples did not vary significantly between patients and remained relatively constant within the prospectively collected samples (Supplementary Material Table S6). ctDNA was quantified in a total of 29 serial plasma samples using a nested-amplification strategy [19, 20] and ddPCR (Supplementary Table S7). Genomic DNA extracted from peripheral blood cells of each patient and cfDNA extracted from healthy donors were used as specificity controls and were negative for all rearrangements assessed. Two distinct personalized biomarkers per patient were used for the detection of ctDNA to overcome issues related to tumor genetic instability and intratumoral heterogeneity. ctDNA was detected by at least one of the two selected biomarkers in all patients at baseline.

### Monitoring treatment response, residual disease and early recurrence with personalized biomarkers and liquid biopsies

#### Patient # 1

**Figure 2:**
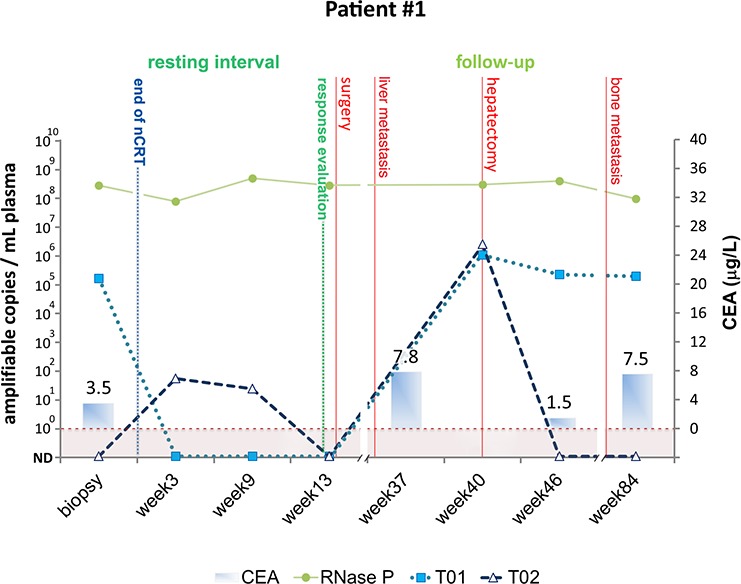
Detection of tumor-specific chromosomal rearrangements in liquid biopsies from Patient #1 Serial blood samples were collected prospectively at diagnosis (biopsy), during the resting interval (weeks 3 and 9), at the time of clinical evaluation of response (week 13), and during follow-up (weeks 40, 46 and 84). The initial treatment for Patient #1 included nCRT and radical surgery for removal of residual tumor. Subsequently, the patient was submitted to a hepatectomy after the diagnosis of liver metastasis and offered palliative treatment after detection of bone metastasis. TaqMan assays amplifying patient specific chromosomal rearrangements (T01 and T02) and a single copy non-rearranged genomic region (RNAse P) were designed to measure ctDNA and total cell-free DNA levels, respectively. ctDNA levels are plotted as relative amplifiable copies/ml of plasma. The horizontal dashed line indicates ctDNA detection limit. CEA levels in μg/ml are plotted as solid bars.

Patient #1 was diagnosed with T3N0 rectal cancer and, at the time of diagnosis, ctDNA was detected at 151,679 amplifiable copies per milliliter of plasma by just one (T01) of the personalized biomarkers (Figure 2). ctDNA levels measured using T01 became undetectable 3 weeks after nCRT completion and remained undetectable until clinical assessment, carried out 13 weeks after nCRT. ctDNA levels measured using T02 were negative at baseline, became marginally positive during the resting period and were also negative at the time of clinical assessment. Clinical and radiological evaluation revealed a marked but incomplete response to nCRT, indicating ctDNA detection was not sensitive enough to detect the presence of residual disease. Pathologic examination of the resected specimen revealed a significant response to nCRT (≤10% viable cancer cells in the resected specimen), which could explain the absence of ctDNA in the samples collected after treatment. This patient was diagnosed with liver metastasis 23 weeks after surgery (elevated CEA and liver nodule at CT scan), which was consistently accompanied by a significant rise in the levels of ctDNA detected at week 40 using both biomarkers. Unfortunately, liquid biopsies collected during the interval between evaluation of nCRT response and the diagnosis of distant metastasis were not available for analysis. This patient underwent radical liver resection and one of the biomarkers (T02) became negative at week 46, while the other (T01) remained abnormal (similar levels to those observed prior to liver resection), suggesting the presence of persistent metastatic disease. However, CEA levels returned to normal shortly after resection of the liver metastases and the patient was considered to be with no evidence of disease. Multiple additional bone metastatic lesions were detected 10 months after liver resection, indicating that biomarker T01 was more sensitive than CEA for the diagnosis of the second metastatic site of disease in this particular patient. Also, levels of biomarker T02 remained negative during follow up, indicating that this rearrangement was exclusively present in the hepatic node and was lost during disease progression. This patient died 21 months after liver resection.

#### Patient #2

**Figure 3:**
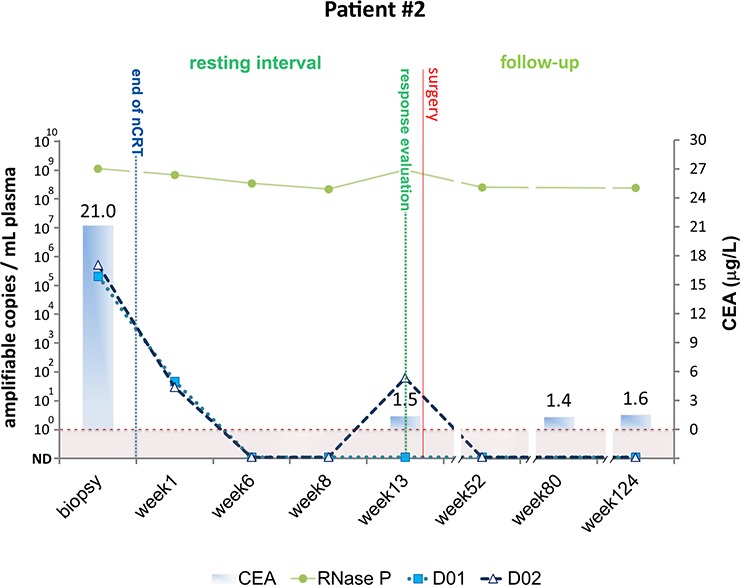
Detection of tumor-specific chromosomal rearrangements in liquid biopsies from Patient #2 Serial blood samples were collected prospectively at diagnosis (biopsy), during the resting interval (weeks 1, 6 and 8), at the time of clinical evaluation of response (week 13), and during follow-up (weeks 52, 80 and 124). The initial treatment for Patient #2 included nCRT and radical surgery for removal of residual tumor. TaqMan assays amplifying patient specific chromosomal rearrangements (D01 and D02) and a single copy non-rearranged genomic region (RNAse P) were designed to measure ctDNA and total cell-free DNA levels, respectively. ctDNA levels are plotted as relative amplifiable copies/ml of plasma. The horizontal dashed line indicates ctDNA detection limit. CEA levels in μg/ml are plotted as solid bars.

Patient #2 was diagnosed with T3N1 rectal cancer and at the time of diagnosis ctDNA was detected at 175,014 and 430,273 amplifiable copies per milliliter of plasma using personalized biomarkers D01 and D02, respectively (Figure 3). ctDNA levels dropped significantly one week after nCRT completion, indicating a good response to nCRT, and became undetectable during the remaining resting period. Marginal levels of ctDNA were detected using D02 biomarker at the time of clinical evaluation, indicating the presence of residual disease after nCRT. Clinical and radiological evaluation revealed incomplete clinical response and pathologic examination of the resected specimen revealed an intermediate response to nCRT (10–50% residual cancer cells). This patient was submitted to radical surgery and is currently with no evidence of recurrent disease after 80 months of follow-up. All liquid biopsies taken during follow-up were negative for the presence of ctDNA.

#### Patient #3

**Figure 4:**
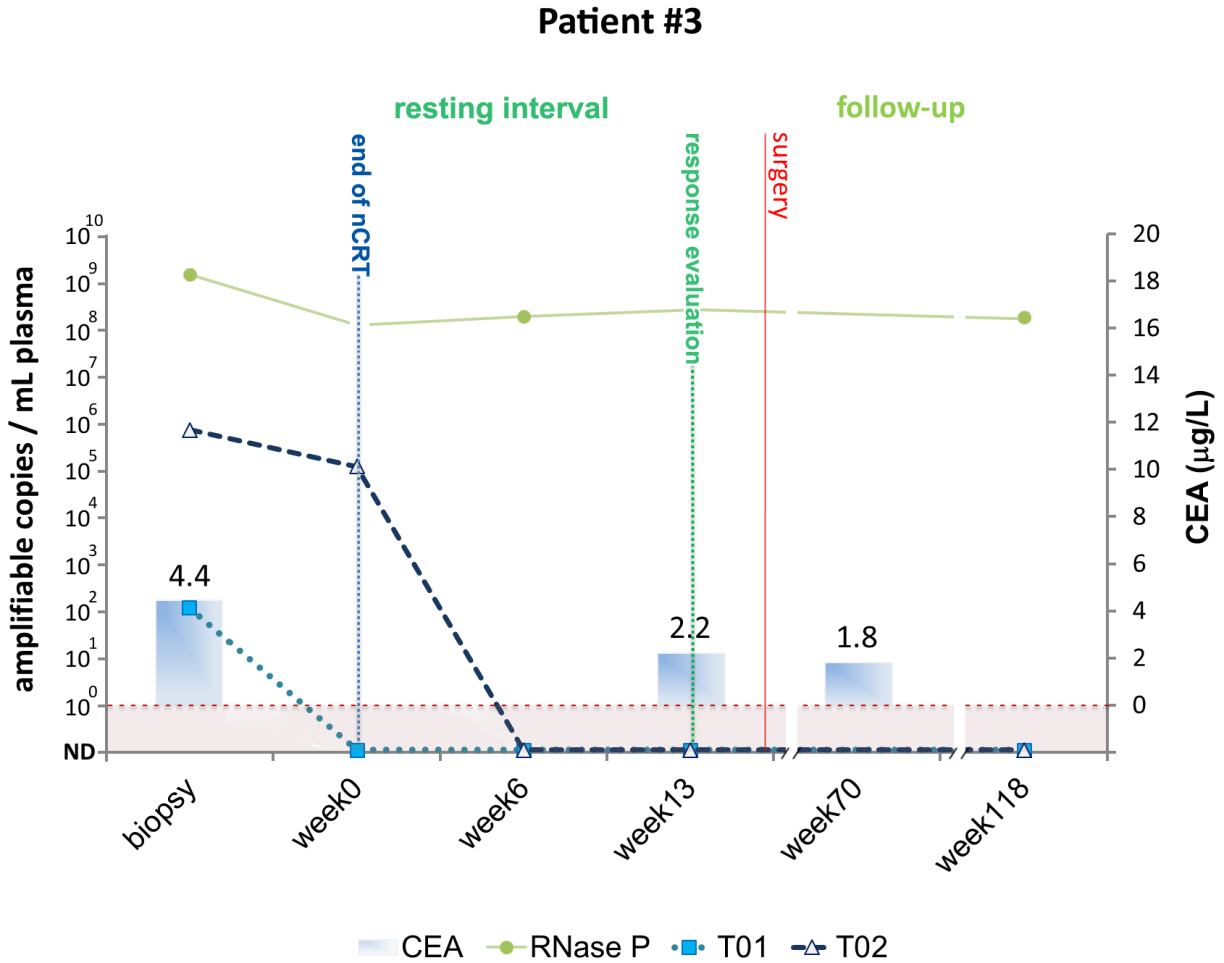
Detection of tumor-specific chromosomal rearrangements in liquid biopsies from Patient #3 Serial blood samples were collected prospectively at diagnosis (biopsy), at the end of nCRT (week 0) during the resting interval (week 6), at the time of clinical evaluation of response (week 13), and during follow-up (weeks 70 and 118). The initial treatment for Patient #1 included nCRT and radical surgery for removal of residual tumor. TaqMan assays amplifying patient specific chromosomal rearrangements (T01 and T02) and a single copy non-rearranged genomic region (RNAse P) were designed to measure ctDNA and total cell-free DNA levels, respectively. ctDNA levels are plotted as relative amplifiable copies/ml of plasma. The horizontal dashed line indicates ctDNA detection limit. CEA levels in μg/ml are plotted as solid bars.

Patient #3 was diagnosed with T3N1 rectal cancer and at the time of diagnosis ctDNA was detected at 658,553 amplifiable copies per milliliter of plasma using one personalized biomarker (T02) and was detected at lower levels using T01 (103 amplifiable copies per milliliter of plasma) (Figure 4). ctDNA levels dropped during nCRT, indicating good response to nCRT, and became undetectable six weeks after nCRT completion. Thirteen weeks from CRT, ctDNA levels remained undetectable. However, clinical and radiological evaluations were insufficient to rule out persistent disease and the patient was referred to radical surgery. Final pathologic examination of the resected specimen revealed pCR and information provided by the absence of biomarkers detection in the peripheral blood of this particular patient, if available to the surgeon at the time of the clinical assessment of response to nCRT, could have helped to avoid unnecessary major surgery and temporary ileostomy. This patient is currently with no evidence of recurrent disease after 60 months of follow-up. Consistently, all liquid biopsies taken during follow-up were negative for the presence of ctDNA.

#### Patient #4

**Figure 5:**
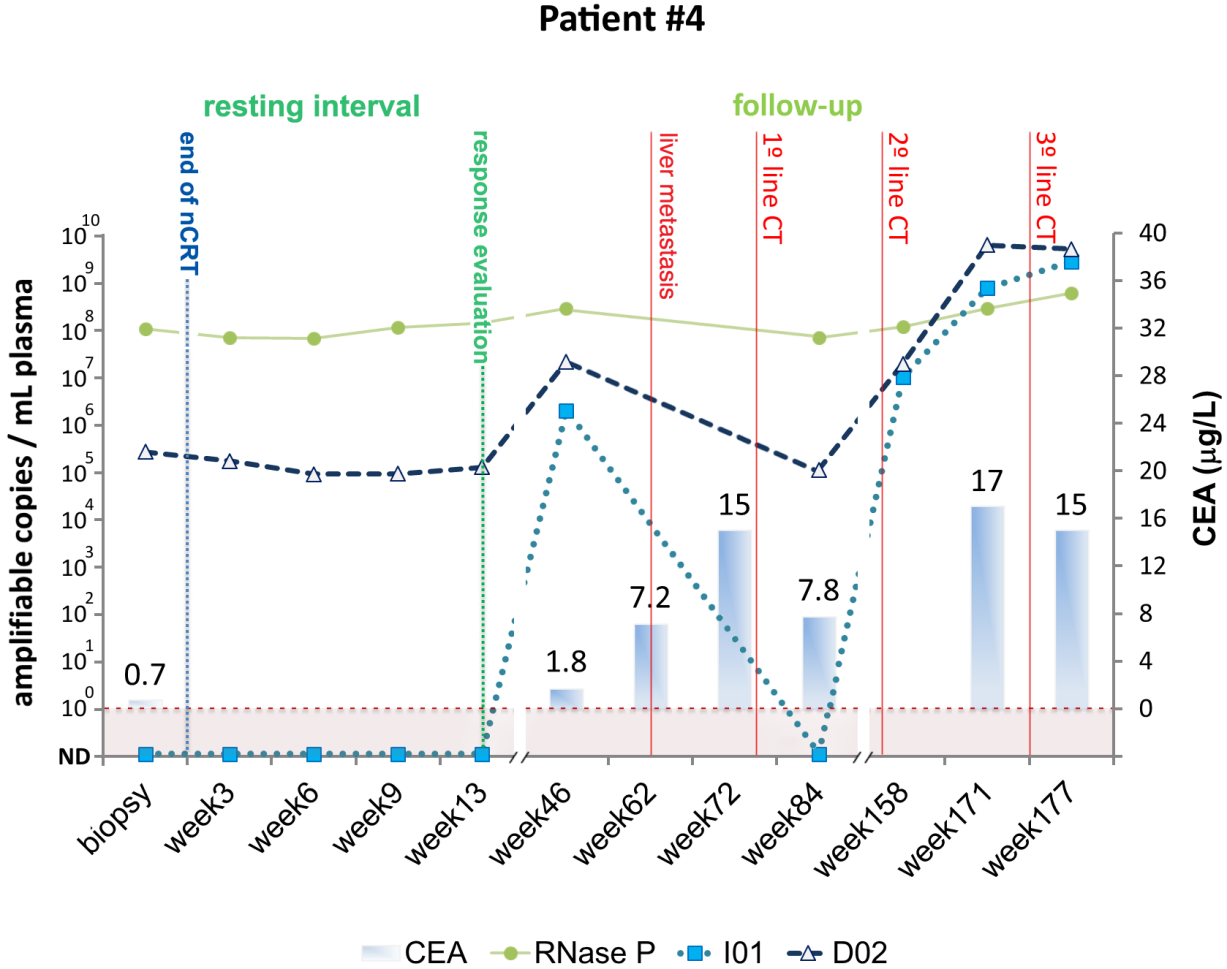
Detection of tumor-specific chromosomal rearrangements in liquid biopsies from Patient #4 Serial blood samples were collected prospectively at diagnosis (biopsy), during the resting interval (weeks 3, 6 and 9), at the time of clinical evaluation of response (week 13), and during follow-up (weeks 46, 62, 72, 84, 158, 171 and 177). The initial treatment for Patient #4 included nCRT and the Watch and Wait Approach. Subsequently, the patient was submitted to different lines of chemotherapy (CT). TaqMan assays amplifying patient specific chromosomal rearrangements (I01 and D02) and a single copy non-rearranged genomic region (RNAse P) were designed to measure ctDNA and total cell-free DNA levels, respectively. ctDNA levels are plotted as relative amplifiable copies/ml of plasma. The horizontal dashed line indicates ctDNA detection limit. CEA levels in μg/ml are plotted as solid bars.

Patient #4 was diagnosed with T3N0 distal rectal cancer and at the time of diagnosis ctDNA was detected at 238,769 amplifiable copies per milliliter of plasma using one of the two biomarkers (D02) (Figure 5). ctDNA levels measured using D02 dropped slightly after completion of nCRT and ctDNA was still detectable at the time of the clinical assessment (13 weeks from nCRT). Thirteen weeks from nCRT completion, clinical and radiological assessment suggested a complete clinical response for the primary tumor and the patient was recommended the Watch and Wait Strategy. Interestingly, 46 weeks from CRT, there was a substantial increase in ctDNA levels detected by both biomarkers, which was not accompanied by increases in CEA levels nor by clinical detection of local or distant recurrence of the disease. Detection of multiple liver metastases was only possible by standard radiological imaging (CT scans) and increased CEA levels at 62 weeks after CRT completion (4 months later). The patient initiated first line chemotherapy and ctDNA levels were assessed at week 84. At this point, ctDNA levels for one biomarker (I01) became undetectable and the other (D02) showed a significant reduction, which reflected a partial response to chemotherapy, confirmed by radiological imaging. Unfortunately, a significant rise in ctDNA levels was again detected for both biomarkers at week 158. The patient initiated second-line chemotherapy and ctDNA levels were monitored using liquid biopsies to determine tumor burden and response to treatment. ctDNA levels continued to increase during follow-up, indicating poor response to treatment and disease progression which was confirmed by radiological imaging. This patient died at 206 weeks from nCRT (48 weeks after initiating second-line therapy) from disease progression.

## DISCUSSION

Patients with locally advanced rectal cancer frequently receive a “one size fits all” approach to treatment, including neoadjuvant therapy followed by radical surgery. Variable degrees of tumor regression are observed after nCRT and a significant proportion of rectal cancer patients develop complete tumor regression. In the absence of residual tumor following nCRT, alternative treatment strategies have been investigated to spare patients from potentially unnecessary adverse outcomes associated with radical surgery [2, 3]. Unfortunately, accurate identification of complete tumor regression remains a significant challenge for colorectal surgeons, endoscopists and radiologists, precluding a more individualized management of rectal cancer patients based on response to nCRT. Surveillance for recurrence after curatively intended nCRT or surgery is also clinically important for rectal cancer patients because early detection of local and systemic recurrences has been shown to be associated with increased patient survival [6, 9].

Even though attempts to standardize clinical and endoscopic findings of a cCR have been made, assessment is highly dependent on individual expertise and is rather subjective. Radiological imaging suffers from inherent limitations of the detection of microscopic residual disease both within the rectal wall and perirectal nodes [7]. Even post-treatment biopsies are often non-informative, particularly when results are negative for residual adenocarcinoma [23]. Finally, CEA is the sole tumor marker that has been shown to be clinically useful in colorectal cancer. However, low specificity and sensitivity of this test precludes its use for individual management decisions in rectal cancer following nCRT [24].

Here, we report the use of personalized biomarkers and liquid biopsies for monitoring treatment response to nCRT and detecting residual disease and early recurrence in patients with locally advanced rectal cancer. For the identification of personalized biomarkers, we used mate-pair low coverage whole genome sequencing and an in-house bioinformatics pipeline designed for the cost-effective identification of a minimal set of chromosomal rearrangements for clinical application that eliminates the need (and cost) to sequence matched germline DNA [22]. Recently, Reinert et al. reported the development of an affordable and robust pipeline for the identification of patient-specific chromosomal rearrangements [20]. However, this pipeline, as opposed to the one used in this work, requires the combination of whole genome sequencing and copy number variation analysis using microarrays, as well as the use of matched tumor and germline DNA, significantly increasing the costs and complexity for implementation in the clinics.

Patient-specific chromosomal rearrangements reported in this work were validated by PCR amplification and breakpoint regions were defined by Sanger sequencing, allowing the development of personalized assays at a cost of ∼US$1,500 per patient in a reasonable timeframe (8 weeks), considering an average interval time of 20 weeks between diagnosis, nCRT and clinical evaluation of response. In total, 4 translocations, 3 intrachromosomal deletions and 1 intrachromosomal inversion were selected as personalized biomarkers for ctDNA detection and, as previously reported, rearrangements occurring in amplified regions of the tumor genome exhibit a higher sensitivity for ctDNA detection [16, 18]. As expected for solid tumors, no identical rearrangement was found in any of the four tumor samples and, although we observed a significant variation in the total number of rearrangements detected in different tumors, all of them were found to have at least 2 bona fide somatic rearrangements that were successfully used as personalized biomarkers for monitoring ctDNA.

We used two distinct personalized biomarkers per patient for ctDNA detection due to limited (3 ml) volume of plasma available. One possible disadvantage of using personalized biomarkers for disease monitoring is the high probability of dealing with passenger rearrangements that may be eventually lost during tumor progression. Unfortunately, there are few studies addressing the stability of personalized biomarkers during tumor progression and, therefore, the exact number of patient-specific chromosomal rearrangements that should be followed to have at least one stable biomarker remains an open question. Most studies suggest the use of at least 3 distinct rearrangements and in these studies consistent results were observed for most, if not all, rearrangements, indicating that the appropriate number is not far from what has been proposed and used in our study [16, 18–20].

A high degree of agreement between measurements obtained with different biomarkers from the same patient was observed only in liquid biopsies collected during disease recurrence and metastatic dissemination, reflecting the low abundance of ctDNA in early disease stages and indicating significant differences in detection sensitivity between different biomarkers, rather than the presence of significant genetic instability and intratumoral heterogeneity. One exception was observed for Patient #1 who was diagnosed with liver metastasis ∼23 weeks after surgery. For this patient, the detection of metastatic disease was accompanied by a significant increase in the levels of ctDNA detected using both personalized biomarkers. Subsequently, this patient was submitted to liver resection and after surgery one of the biomarkers became negative (T02), while the other was detected at similar levels to those observed before surgery. A second metastatic lesion was detected in the bone after liver resection and levels of T02 biomarker remained negative even during palliative treatment, indicating that this rearrangement was exclusively present in the hepatic node and was lost during disease progression.

Noteworthy, the rearrangement that was consistently detected during disease progression and dissemination involved the TP53 Binding Protein 2 (TP53BP2) intragenic region. TP53BP2 enhances TP53-mediated transcriptional activation and has been proposed to play a key role in the response to DNA damage and checkpoint signaling during mitosis [25]. Although no further analysis was carried out to address the functional role of this genetic alteration during tumor progression, it is very tempting to speculate that this particular rearrangement involving TP53BP-2 constitutes a driver genetic event associated with disease progression and dissemination after nCRT.

In this proof-of-concept study, we observed that ctDNA levels measured using personalized biomarkers and liquid biopsies were efficient in monitoring tumor burden and dynamics in response to oncological intervention, and in the early detection of incipient recurrence. As expected for locally advanced tumors, ctDNA was detected at relatively low levels at baseline in patients with T3 rectal tumors, increasing during tumor progression. These results are in agreement with a recent study published by Bettegowda et al. in which ctDNA was detected in 73% of the patients with localized colorectal tumors and a direct correlation between ctDNA concentration and disease stage was observed [12]. Detection of ctDNA at baseline in patients with T2 rectal tumors was not successfully achieved (data not shown).

We showed that personalized biomarkers and liquid biopsies were successfully used to monitor treatment response to nCRT. All patients included in our study presented significant tumor regression (TRG3–4), which was directly reflected by a decrease in ctDNA levels during the resting period. Although a recent study have demonstrated the utility ctDNA as an early marker of therapeutic response to first-line adjuvant chemotherapy, all patients included in this study had metastatic colorectal cancerand higher levels of ctDNA [26]. Therefore, to the best of our knowledge, our study was the first one to assess the utility of early changes in ctDNA levels to monitor response to neoadjuvant therapy in rectal cancer patients with locally advanced disease.

Personalized biomarkers were also successfully used to detect early metastatic disease and disease progression, preceding increases in CEA levels and radiological diagnosis even among patients with excellent primary tumor response to nCRT. Good results were also obtained when assessing tumor response to first and second line adjuvant therapy in our patients. Our results are in agreement with a recently published manuscript in which the authors have demonstrated that ctDNA detection enabled efficient monitoring of tumor burden and early detection of recurrence following colorectal cancer surgery [20]. Our promising findings may have a significant impact in clinical management of patients with rectal cancer. First, earlier detection of disease progression may result in a higher proportion of patients being diagnosed with curable metastatic disease. Second, this information may also be relevant for the early change of treatment regimen, particularly among patients undergoing palliative treatment where different lines of therapy are currently available. Also, even though none of our patients developed local recurrences, these biomarkers may also provide relevant information among patients in this setting, potentially increasing the possibility of salvage therapies. Finally, these biomarkers may ultimately be incorporated in routine follow-up of rectal cancer patients, minimizing the need for radiological imaging with significant impact in follow-up cost burden and invasiveness.

Unfortunately, ctDNA detection proved insufficiently sensitive to rule out the presence of microscopic residual disease after nCRT completion. Although, ctDNA levels were negative in the patient with complete pathologic response at the time of clinical assessment and this information, if available to surgeons, could have prevented unnecessary surgery, ctDNA levels were also negative among patients presenting incomplete clinical response with significant tumor regression (≤10% of tumor cells present in the resected specimen. In this case, non-operative management based on the information of ctDNA levels alone would have been inappropriate. In contrast, the patient with complete clinical response presented persistent positivity for ctDNA, which, in this particular case was not associated with the presence of residual disease. This patient did not present local disease recurrence during follow up but was lately diagnosed with distant metastases by radiological imaging. Still, with this information upfront after nCRT completion, aggressive systemic chemotherapy could have been offered to improve the oncological outcomes in this patient.

In conclusion, the implementation of personalized biomarkers and liquid biopsies in the management of patients submitted to nCRT may improve the accuracy of clinical and radiological assessment of patients submitted to nCRT and offer, in the near future, a concrete opportunity for a more personalized treatment of rectal cancer patients. Although our initial experience is encouraging, our study has important limitations, specially concerning the small number of patients and biomarkers analyzed and the limited volume of plasma available for ctDNA detection. These limitations certainly have a direct impact on the determination of the accuracy in the assessment of tumor response and presence of residual disease, as well as in the estimate of the percentage of patients that will have informative baseline results. Therefore, replication in a larger prospective cohort using larger volumes of plasma and higher numbers of personalized biomarkers per patient is necessary to introduce this strategy into clinical practice.

## MATERIALS AND METHODS

### Initial patient assessment and treatment

Four patients with cT3N0–1M0 biopsy-proven rectal adenocarcinoma, located up to 7 cm from the anal verge were included in this IRB-approved study (Hospital Alemão Oswaldo Cruz, São Paulo, SP, Brazil). Baseline staging included magnetic resonance imaging and/or endorectal ultrasound for local assessment and abdominal and chest computed tomography for systemic staging. Briefly, all patients underwent nCRT including 50.4–54 Gy of radiation and 5FU-based chemotherapy as described elsewhere [6].

### Assessment of response to nCRT and follow-up

Patients were assessed uniformly after 12–13 weeks from nCRT completion using identical clinical, endoscopic and radiological parameters used for primary assessment. Patients with clinical or radiological evidence of persistent cancer were referred to immediate radical surgery, including total mesorectal excision and were followed every three months for the first 2 years and every 6 months thereafter. Patients with no evidence of residual disease were not immediately operated and were enrolled in a strict follow up protocol (Watch and Wait strategy) as described elsewhere [21]. Adjuvant chemotherapy was offered only for patients with pathologic evidence of nodal metastases in the resected specimen (ypN+). None of the patients with complete clinical response received adjuvant chemotherapy. Information on the levels of biomarkers was not available to the surgeons or medical oncologists and was not used for management decisions.

### Tumor samples

Tumor samples were snap-frozen in liquid nitrogen and stored at −80°C immediately after endoscopic biopsies. Prior to DNA extraction, all fragments were stained with hematoxilin-eosin and macrodissected for the presence of at least 80% adenocarcinoma. Tumor genomic DNA was extracted with Trizol (Life Technologies) using a protocol provided by the manufacturer for simultaneous extraction of DNA and RNA. Peripheral blood cells were collected from each patient and genomic DNA, extracted with standard phenol-chloroform protocol, was used as matched normal DNA to confirm the somatic origin of the patient-specific chromosomal rearrangements.

### Plasma samples

Blood samples (10–12 ml) collected in tubes containing EDTA were processed within 2 hours after collection. Samples were centrifuged twice to separate the plasma from the peripheral-blood cells and from cellular debris and were stored at −80°C. Circulating free DNA (cfDNA) was extracted from 3ml aliquots of plasma using the QIAamp MinElute Virus Vacuum Kit (QIAGEN). cfDNA was eluted into 125 μL and stored at −80°C

### Identification of patient-specific chromosomal rearrangements

DNA sequencing was performed using the SOLiD platform (Life Technologies). Mate-pair libraries were generated starting from 5 μg of tumor genomic DNA sheared into 0.6–1.0 kb fragments, according to manufacturer's instructions. Sequence data were mapped against the hg19/GRCh37 human genome reference sequence using Bioscope (Life Technologies). Mapped sequences selected for further analysis were required to match the reference genome uniquely with a mapping quality greater than or equal to 20 (Q > = 20). Selected sequences were analyzed for aberrant mate-pair spacing and orientation using ICRmax [22]. For the identification of intrachromosomal deletions, read pairs mapping on the same chromosome within distances larger than 4Kb were selected and submitted to ICRmax. Interchromosomal rearrangements and intrachromosomal deletions and inversions were selected when reported by at least 3 independent sequence-pairs and validated by PCR amplification and Sanger sequencing across the breakpoint region using tumor and matched normal DNA as templates to confirm their somatic origin. Primer sequences used for validation are provided as Supplementary Material Table S1.

### Detection of circulating tumor DNA

Total circulating DNA was measured by absolute quantification using the RNaseP Copy Number Reference Assay (Life Technologies). Circulating tumor DNA was detected using the QX200 Droplet Digital PCR (ddPCR) system according to the manufacturer's instructions (Bio-Rad). Increasing volumes of cfDNA (2–4 μl) were used for the initial quantification to check for the presence of PCR inhibitors and determine the maximum amount of cfDNA that could be used in the ddPCR assays. For monitoring ctDNA, a previously described nested PCR approach [19, 20] was adopted to maximize sensitivity. Briefly, an initial multiplex PCR, with primers flanking the breakpoints of 2 patient-specific chromosomal rearrangements and a single copy control genomic region (H1RNaseP) were used to pre-amplify cfDNA for 20 cycles using SsoFast Supermix (Bio-Rad). In this initial amplification 3–7 μl of cfDNA were used per reaction and at least 8 independent reactions were performed for each plasma sample and were grouped for ctDNA quantification using the merge function of the QuantaSoft Software (Bio-Rad). A total of 3 μl of the multiplex pre-amplification reaction was then used as template for ddPCR reactions in which each patient-specific chromosomal rearrangements were analyzed separately. The size of the initial amplicons were kept to <200bp due to the highly fragmented nature of the cfDNA. Primers placed internally to the initial amplicon and a labeled DNA Taqman probe (6-FAM-MGB) crossing the breakpoint region were designed using PrimerExpress software (Applied Biosystems) and sequences are provided as Supplementary Material Table S2. Linearity and sensitivity of the patient-specific assays for ctDNA detection were assessed using a six-point dilution series of tumor DNA (4000, 1000, 250, 62.5, 15.6 and 3.9 genomes) in a constant pool of 20.000 genomes of normal DNA. Genomic DNA extracted from peripheral blood cells of each patient and cfDNA extracted from healthy donors were used as specificity controls. To ensure the accuracy of the results, a minimum of 10,000 acceptable droplets per reaction were required for quantification using the QuantaSoft software (Bio-Rad). Samples yielding a minimum of 3 positive droplets from 10–15,000 droplets analyzed were scored as positive.

## SUPPLEMENTARY TABLES


