# Supplementary material for: The use of personalized biomarkers and liquid biopsies to monitor treatment response and disease recurrence in locally advanced rectal cancer after neoadjuvant chemoradiation
Source: Oncotarget. 2015 Oct 6;6(35):38360–71. doi: 10.18632/oncotarget.5256 (PMC4742005; doi:10.18632/oncotarget.5256)
Supplement: Supplementary file 1 [file oncotarget-06-38360-s001.pdf]

SUPPLEMENTARY TABLES

Supplementary Table S1: Patient-specific chromosomal rearrangements were identified by computational analysis and validated by PCR and Sanger Sequencing. Primers used for validation and sequencing are listed

Patient	SV	Sequences	
		Primer 1 (5′ → 3′)	Primer 2 (5′ → 3′)
1	T01	GGCTCCTTGTCCGTATCAC	AAGAGTGCAGTTGAAGTCCTG
1	T02	CACTAATGGGGAAACCGTTG	TGCCCTCCAATTAACCATTC
1	T03	TGAATTCCACCGCAAACGAG	GGTACAGTCCATTCATGTTGAG
1	T05	GAGGTAATGGCCAGGTAAAG	CTTCTTCCTTTAAGCCGATGC
1	T06	CTGTAGTTCGCCAATCCTG	CTCTCCTGTGGAAGTGATTG
1	T07	AAGGAGCAAACAAGCACCTC	CTCCTGACTCCTGAAAGCTG
1	T08	TGCATTGGAGGCTAATCACATG	GGCAAGAACAAGAGGCAAAC
1	T11	ATTTCTGGGCATTCCTGAGC	CTGCAATTCCACGTCATCTC
1	T12	ATATCGCCCATTGCAATCTC	AGTGCAGTGCTTGTCACAG
1	T13	TGTCAAGTGGAAAGGTAAGC	GAGGTCAGCAGGTCATCC
2	T01	CTTTCACGTGGAACCAGTTTG	ACCTCTGGGCACTGAAAAAG
2	T03	TTCCCAAAATATACCCAATTACTG	TGTGGTGTTTGTCTTCAGTGG
2	T04	TTCACAGAGCACAAGGAGATCC	GCCAGTAATTGGGTATATTTTGG
2	D01	AACCACATTGTTGAGGAGAGCC	ACGCTTGGACTATTGGCTTCC
2	D02	CTCAGCTCTGGACATCTCTATGC	ACGTCCTGTCCCCAAGAAC
2	D04	AATCCTCCACCCAATTCATAAGC	AGTTGTTCATAGGGTTGCAATGG
2	D06	CAGCCAGCAGTTTTCTGTTCC	AGAATGTTCTGGTCTGGTCTCG
2	D07	GAAAACTGCATTGCATGTGGTC	TAACATGCCCTGTTGTCCTGAG
2	D09	GTGGACTGCTGTCGGGTAAC	TTCCTCTGTACCTGGCCATTG
2	D11	CTCATCTGGAGCCTTGTTC	AGACAGGAAAGGACACCAAGC
3	T01	CAATTCGCGTATCTGCAAAGAG	ATTACGCAGCAGCATGTGAC
3	T02	GTTTAGATGCGCAGGGAGAG	CTGGAAAGCCCTAAGTGTGG
3	T03	TGATTTTGGTGACAGTATCATGG	TGGCTAGTAGATCTCTCTCC
4	I01	CCATTTCCTAGCCAAACCTG	GCATCCACTAGGGGTCTTAG
4	D02	CCTTTCCCGTTACACACCAC	GGCCAGTCTGAATGAGGATG
4	I03	CTGGGAAATGCAGGTGTAGG	ATGTTGGAGGCCTTTCTTCC
4	T01	CAGCATGCTGATGAGGCTTG	CGAGAATTACTGCCATGAAGC
4	T02	ACGATGTACCGCTCAAGTCC	TCTGCCCAGATAAGCTACAGG
4	T03	TTCGAAAACGGTTGTTTGTGG	GGAGCCATGGCAGAAGAAC

Supplementary Table S2: Primers and Probes designed for the detection of patient-specific chromosomal rearrangements in liquid biopsies

Patient	SV	External oligonucleotides[a]		amplicon AB	Internal oligonucleotides[b]		Amplicon[c] CD	MGB Probe[d]
		primer A (5′→ 3′)	primer B (5′→ 3′)		primer C (5′→ 3′)	primer D (5′→ 3′)		
1	T01	GCCAGAAGGTCCCAGA CACTG	AAGAGTGCAGTTGAAG TCCTG	115bp	GAGTATACAGAAATGGG TGAGTGC	TGCAGTTGAAGTCCTG GATTAAGG	80bp	CAGCAATGTGAAGTAAA
1	T02	AGGGAAGAGCTCATGC CAGC	GTTTGATCTTGACAATG GTGCTG	143bp	ATCATTTCTTAGCATCAG CTATGC	TGACAATGGTGCTGTGT AAACGTC	99bp	CCATGAgagaGAGAAG
2	D01	AACCACATTGTTGAGGA GAGCC	GAATTTTGTTCTGGGCA TGAGC	133bp	AGAGCCATACAATACAG TCAGG	CTGGGCATGAGCATGA CTGTAC	107bp	TCTGGTCCATCTTTC
2	D02	TCAATCAAAATGGGCCT TTCAC	GACGTCCTGTCCCCAA GAAC	179bp	CTCAGCTCTGGACATCTCT ATGC	ACAGTCACTGCAGATGT GATTCG	151bp	TCAGCATGTACCTTGTC
3	T01	GACTCAGAATGTATTCA GTCG	ATTACGCAGCAGCATGT GAC	159bp	TGGATGCTGAACTCTG GTGTTAGG	ACGCAGCAGCATGTGA CTGTAG	130bp	TACCAGGCAAAGTGG
3	T02	AGTTTAGATGCGCAGGG AGAG	ACTACAGTACTCCTGAG AATGC	153bp	GCTGTCCCTGCCACAAT TATGTCA	CTCCTGAGAATGCACTG AAGAGAAG	113bp	ACGGCACAGTATTGG
4	I01	CTCACACAGTTTTCTGT TGTTGG	AAACACAGCATATATAG GGTCCAG	229bp	ATGCGCCAATCAGCACT CTG	GGTCCAGCAGTATCCAT GATTTCA	154bp	CTAGAGGTTGGATAAACT
4	D02	CATTTGCCACGCTTTGT GAC	GTCTCAAACTCCTCTGC TCAAG	143bp	GACAGGCCCTTTCCCG TTAC	CCTCAGCCTCCCAAATT GTTG	93bp	ACACCTGCCCTGTGAA

Abbreviations: (**SV**) Structural Variation; (**D**) Deletion; (**I**) Inversion; (**T**) Translocation.

[a]Sequences for external oligonucleotides used for pre amplification;

[b]Internal oligonucleotides and probes (ddPCR) used for the detection of ctDNA by ddPCR.

[c]Amplicon: size of PCR product in base pairs;

[d]MGB Probe: complementary to the breakpoint sequence. Lowercase letters represent bases inserted during the chromosomal rearrangement and that are not present in the human genome reference sequence.

Supplementary Table S3: Whole genome sequencing and mate-pair mapping data

Patient	Reads generated	Nucleotides generated	Reads mapped	Reads mapped [a] Q > =20	Sequence coverage	Physical coverage [b]
1	393,756,912	19,687,845,600	322,877,464 (82%)	256,652,253 (65%)	4.07x	19x
2	1,035,604,016	51,780,200,800	788,793,614 (76%)	560,355,119 (54%)	8.65x	60x
3	385,789,584	19,289,479,200	305,832,709 (79%)	242,963,348 (63%)	3.70x	18x
4	425,460,416	29,782,229,120	350,515,565 (82%)	247,758,837 (58%)	4.83x	13x

[a]Only sequences showing reliable mapping Q > =20 were selected. Q = Quality of mapping. Q >= 20 means the alignment has a 99% chance of being correct.
[b]Physical coverage is calculated using only mate-pairs that show correct orientation and expected distance.

Supplementary Table S4: Patient-specific chromosomal rearrangements identified using ICRmax

Patient	SV	Number of reads	Chromosomal region[a]				Amplicon[b]	Status[c]
			Chr A	Position A	Chro B	Position B		
1	T01	9	17	35126102	1	223988874	215bp	TRUE
1	T02	6	8	40477264	2	152374971	536bp	TRUE
1	T03	5	2	122159723	8	127649237	577bp	TRUE
1	T04	2	8	-73783752	11	40398761	–	not evaluated
1	T05	5	2	30169357	8	-127636126	458bp	TRUE
1	T06	7	7	127318226	14	53067743	524bp	TRUE
1	T07	5	11	-10847237	1	16502493	707bp	TRUE
1	T08	3	14	53067736	7	127318227	644bp	TRUE
1	T09	3	8	74841613	2	-195681531	–	not evaluated
1	T10	4	1	53615346	5	167865176	–	not evaluated
1	T11	5	1	-119766787	17	35387373	954bp	TRUE
1	T12	3	4	-156768839	11	87895463	262bp	TRUE
1	T13	4	8	74814865	2	128394833	–	FALSE
1	D01	6	1	58338901	1	104111466	–	not evaluated
1	D02	11	1	61444642	1	155822926	–	not evaluated
1	D03	5	1	155870136	1	222019855	–	not evaluated
1	D04	5	2	221000576	2	122992576	–	not evaluated
1	D05	19	6	53928657	6	53934870	–	not evaluated
2	T01	4	14	85145043	8	74493953	1016bp	TRUE
2	T02	3	13	102813872	2	-135875336	–	not evaluated
2	T03	8	22	29065971	2	238177710	~650bp	TRUE
2	T04	5	2	238177423	22	29065747	~760bp	TRUE
2	D01	9	3	60342822	3	60388671	251bp	TRUE
2	D02	11	4	185436839	4	185913887	150bp	TRUE
2	D03	5	X	6831906	X	6908289	–	Not evaluated
2	D04	7	1	210077275	1	210086003	–	FALSE
2	D05	5	2	106879453	2	106885973	–	Not evaluated
2	D06	6	5	9207770	5	9435181	213bp	TRUE
2	D07	5	5	58772843	5	58858549	492bp	TRUE
2	D08	5	6	32630672	6	32723496	–	Not evaluated
2	D09	14	6	77016657	6	77029247	–	FALSE
2	D10	5	9	23362336	9	23377749	–	not evaluated
2	D11	9	18	51205947	18	51210677	–	FALSE

(*Continued*)

Patient	SV	Number of reads	Chromosomal region[a]				Amplicon[b]	Status[c]
			Chr A	Position A	Chro B	Position B		
3	T01	5	8	93421404	6	13191227	778bp	TRUE
3	T02	3	12	3607669	6	145470434	648bp	TRUE
3	T03	4	6	65543456	1	-62053749	1011bp	TRUE
3	T04	2	19	40086883	14	-65447676	–	not evaluated
3	D01	7	3	121356366	3	121382720	–	not evaluated
3	D02	9	5	8591553	5	8598032	–	not evaluated
3	D03	6	5	54798967	5	125145693	–	not evaluated
3	D04	9	10	79265480	10	88087970	–	not evaluated
3	D05	10	10	79265512	10	88092435	–	not evaluated
3	D06	7	16	6109988	16	6116508	–	not evaluated
3	D07	5	16	6298413	16	6312549	–	not evaluated
3	D08	7	16	6329791	16	6909675	–	not evaluated
3	D09	6	16	78371178	16	78384996	–	not evaluated
3	D10	7	20	14897971	20	14938767	–	not evaluated
3	D11	5	20	14926929	20	15109439	–	not evaluated
4	I01	32	12	1819278	12	-1870423	293bp	TRUE
4	D02	4	17	39823076	17	39837384	742bp	TRUE
4	I03	4	17	39113895	17	-39119434	–	FALSE
4	T01	5	3	7252285	5	110411594	657bp	TRUE
4	T02	4	13	21742463	11	108585788	–	FALSE
4	T03	2	6	-93096822	3	187729086	–	FALSE

Abbreviations: (**SV**) Structural Variation; (**D**) Deletion; (**I**) Inversion; (**T**) Translocation; (**ChrA**) Chromosome A; (**ChrB**) Chromosome B.

[a]Chromosomal regions were based on the human genome reference sequence (hg19), negative signal indicates that sequence aligned to the minus strand. Chromosomal coordinates correspond to breakpoint coordinates.

[b]Amplicon: PCR fragment size in base pairs;

[c]Status: (TRUE) Validated patient-specific chromosomal rearrangements; (FALSE) Not validated by PCR and Sanger sequencing

Supplementary Table S5: List of validated patient-specific chromosomal rearrangements

Patient	inter-chromosomal variation			intra-chromosomal variation			total SV validated
	total identified	evaluated	validated	total identified	evaluated	validated	
1	13	10	9	5	—	—	9
2	4	3	3	11	7	4	7
3	4	3	3	11	—	—	3
4	3	3	1	3	3	2	3

Supplementary Table S6: cfDNA quantification using ddPCR

Patient	Sample	Plasma (mL)	Elution (µL)	Assay	total of analyzed droplets	copies/mL plasma[a]
1	biopsy	2	125	RNaseP	14318	396
1	week 3	3	125	RNaseP	14563	271
1	week 9	3	125	RNaseP	14191	813
1	week 13	1,2	50	RNaseP	14587	1104
1	week 40	3	125	RNaseP	14367	31389
1	week 46	3	125	RNaseP	11581	708
1	week 84	3	125	RNaseP	13912	208
2	biopsy	3	125	RNaseP	11422	4063
2	week1	3	125	RNaseP	16936	2083
2	week6	3	125	RNaseP	9853	917
2	week8	3	125	RNaseP	16170	1750
2	week13	3	125	RNaseP	11169	6583
2	week52	3	125	RNaseP	12428	1333
2	week124	3	125	RNaseP	9308	729
3	biopsy	3	125	RNaseP	15973	3056
3	week 0	3	125	RNaseP	14688	722
3	week 6	3	125	RNaseP	13984	417
3	week 13	3	125	RNaseP	15779	889
3	week 118	3	125	RNaseP	14143	667
4	biopsy	3	125	RNaseP	16714	500
4	week 3	3	125	RNaseP	10562	333
4	week 6	3	125	RNaseP	17976	500
4	week 9	3	125	RNaseP	17090	667
4	week 13	3	125	RNaseP	9963	167
4	week 46	3	125	RNaseP	15517	917
4	week 84	3	125	RNaseP	11399	771
4	week 158	3	125	RNaseP	17239	729
4	week 171	3	125	RNaseP	19238	1000
4	week 177	3	125	RNaseP	16195	2028
4	week 177	3	125	RNaseP	16195	2028

[a]number of copies per reaction was calculated by Quantasoft software and normalized to cfDNA volume added in the first PCR step.

Supplementary Table S7: ctDNA quantification using ddPCR

Patient	Sample	Analyzed cfDNA volume (µL)	Assay I			Assay II			ctDNA detection[a]
			SV	analyzed droplets	Positive droplets	SV	analyzed droplets	Positive droplets	
1	biopsy	56	T01	105351	10809	T02	72942	2	YES
1	week 3	112	T01	211725	2	T02	196531	7	YES
1	week 9	105	T01	220455	0	T02	222770	3	YES
1	week 13	28	T01	88194	0	T02	83943	0	no
1	week 40	12	T01	53415	13811	T02	48624	24743	YES
1	week 46	48	T01	77174	9097	T02	98049	0	YES
1	week 84	48	T01	86158	8930	T02	82655	1	YES
2	biopsy	112	D01	231067	27137	D02	228797	60518	YES
2	week 1	112	D01	216201	3	D02	213936	4	YES
2	week 6	112	D01	245592	2	D02	241461	0	no
2	week 8	112	D01	220527	2	D02	229876	0	no
2	week 13	112	D01	216493	0	D02	252034	10	YES
2	week 52	112	D01	212009	2	D02	250350	1	no
2	week 124	112	D01	235582	1	D02	216205	0	no
3	biopsy	45	T01	124679	7	T02	137620	41562	YES
3	week 0	60	T01	170754	0	T02	178569	10442	YES
3	week 6	96	T01	209758	1	T02	256007	1	no
3	week 13	100	T01	295679	1	T02	293765	1	no
3	week 118	96	T01	226729	0	T02	231798	1	no
4	biopsy	44	I01	147158	0	D02	155873	14466	YES
4	week 3	55	I01	166120	1	D02	168166	12652	YES
4	week 6	48	I01	75989	0	D02	89458	4363	YES
4	week 9	56	I01	104309	0	D02	109229	6250	YES
4	week 13	90	I01	240070	0	D02	228506	12840	YES
4	week 46	54	I01	124984	110813	D02	132785	132784	YES
4	week 84	105	I01	162833	0	D02	185962	12800	YES
4	week 158	112	I01	223646	223478	D02	241682	241681	YES
4	week 170	40	I01	139513	106952	D02	140040	140039	YES
4	week 175	40	I01	118251	117683	D02	119466	119460	YES

[a]Yes = at least one of evaluated assay was detected in the sample.
